# Data on electronic structures for the study of ligand effects on the zirconocene-mediated trimethylene carbonate polymerization

**DOI:** 10.1016/j.dib.2018.09.042

**Published:** 2018-09-19

**Authors:** Jitrayut Jitonnom, Wijitra Meelua

**Affiliations:** Division of Chemistry, School of Science, University of Phayao, Phayao, 56000 Thailand

## Abstract

The data presented in this paper are related to the research article entitled “Effect of ligand structure in the trimethylene carbonate polymerization by cationic zirconocene catalysts: A “naked model” DFT study” (Jitonnom and Meelua, 2017) [1]. In this data article, we present 3D molecular information of 29 zirconocene catalysts that differ in electronic and steric properties. The data contains all cationic species along the initiation and first propagation step of the polymerization, which are provided in a PDB format that can be used for further studies.

## Specifications table

**Table:** 

Subject area	*Chemistry*
More specific subject area	*Organometallic chemistry*
Type of data	*PDB files*
How data was acquired	*Density functional theory calculations with Gaussian*
Data format	*Raw*
Experimental factors	*The dataset of 29 catalyst structures were generated from density functional theory (DFT) calculations*
Experimental features	*Geometry optimization with B3LYP functional.*
	*Effective core potential double-*ζ *basis set (LANL2DZ) was used for zirconium atom, while a double-*ζ *basis set (6–31G(d)) for all non-metal atoms.*
Data source location	*Bangkok, Thailand*
Data accessibility	*Data are supplied with this article.*
Related research article	*[1] Jitonnom J., Meelua W. Effect of ligand structure in the trimethylene carbonate polymerization by cationic zirconocene catalysts: A “naked model” DFT study. J. Organomet. Chem. 2017 841: 48–56.*

## Value of the data

• The 29 zirconocene catalysts could serve as a structural database for the researchers in screening new catalyst before experiment.
• Method and basis set employed in this data article can be used as a reference for future studies
• The PDB coordinates of the catalyst models can serve as starting points for further electronic structure calculations.

## 1 Data

The data described in this paper provides information for the electronic structures for five cationic species, *i.e.* isolated cation (**Cation**) TMC-activated complex (**Complex**) reactant (**Re**) transition state (**TS**) and product (**Pr**) (see Ref. [1] for more details) in the initiation and first propagation step of cationic ring-opening polymerization (CROP). The dataset of 29 catalyst structures were generated from density functional theory (DFT) calculations [2], [3], [4]. Atomic coordinate files for each species of all catalysts were also provided in PDB format.

## 2 Experimental design, materials, and methods

All geometries were fully optimized at the B3LYP level of theory using Gaussian 09 program [5]. The effective core potential double-ζ basis set (LANL2DZ) [6] was used for Zr atom, while a double-ζ basis set (6–31G(d)) for all non-metal atoms (C, H, O, F, Si, and Ge). This DFT/mixed basis set method has been shown to reproduce the X-ray structure [7] and has been widely applied in the field of transition metal complexes [7], [8], [9], [10], [11], [12], [13], [14], [15], [16]. Frequency calculations were performed on all optimized structures to confirm the nature of stationary points as minima or transition states.

## References

[1] Jitonnom J., Meelua W. (2017). Effect of ligand structure in the trimethylene carbonate polymerization by cationic zirconocene catalysts: a “naked model” DFT study. J. Organomet. Chem..

[2] Parr R.G., Yang W. (1994). Density-Functional Theory of Atoms and Molecules.

[3] Kohn W., Sham L.J. (1965). Self-consistent equations including exchange and correlation effects. Phys. Rev..

[4] Jitonnom J., Sattayanon C., Kungwan N., Hannongbua S.A. (2015). DFT study of the unusual substrate-assisted mechanism of *Serratia marcescens* chitinase B reveals the role of solvent and mutational effect on catalysis. J. Mol. Graph. Model..

[5] Frisch M.J., Trucks G.W., Schlegel H.B., Scuseria G.E., Robb M.A., Cheeseman J.R., Scalmani G., Barone V., Mennucci B., Petersson G.A., Nakatsuji H., Caricato M., Li X., Hratchian H.P., Izmaylov A.F., Bloino J., Zheng G., Sonnenberg J.L., Hada M., Ehara M., Toyota K., Nakatsuji R., Hasegawa J., Ishida M., Nakajima T., Honda Y., Kitao O., Nakai H., Vreven T., Montgomery J.A., Peralta J.E., Ogliaro F., Bearpark M., Heyd J.J., Brothers E., Kudin K.N., Staroverov V.N., Kobayashi R., Normand J., Raghavachari K., Rendell A., Burant J.C., Iyengar S.S., Tomasi J., Cossi M., Rega N., Millam J.M., Klene M., Knox J.E., Cross J.B., Bakken V., Adamo C., Jaramillo J., Gomperts R., Stratmann R.E., Yazyev O., Austin A.J., Cammi R., Pomelli C., Ochterski J.W., Martin R.L., Morokuma K., Zakrzewski V.G., Voth G.A., Salvador P., Dannenberg J.J., Dapprich S., Daniels A.D., Farkas O., Foresman J.B., Ortiz J.V., Cioslowski J., Fox D.J. (2009). Gaussian 09 Revision A.02.

[6] Yang Y., Weaver M.N., Merz K.M. (2009). Assessment of the "6-31+G** + LANL2DZ" mixed basis set coupled with density functional theory methods and the effective core potential: prediction of heats of formation and ionization potentials for first-row-transition-metal complexes. J. Phys. Chem. A.

[7] Jitonnom J., Meelua W. (2014). Effects of silicon-bridge and π-ligands on the electronic structures and related properties of dimethyl zirconocene polymerization catalysts: a comparative theoretical study. Chiang Mai J. Sci..

[8] Liu Y., Liu Y., Drew M.G.B. (2010). Correlation between regioselectivity and site charge in propene polymerisation catalysed by metallocene. Struct. Chem..

[9] Zhang C.-G., Yu S.-Y., Zhang L., Li H., Wang Z.-X. (2015). DFT mechanistic study of the H_2_-assisted chain transfer copolymerization of propylene and p-methylstyrene catalyzed by zirconocene complex. J. Polym. Sci. Part A: Polym. Chem..

[10] Valente A., Zinck P., Mortreux A., Visseaux M., Mendes P.J.G., Silva T.J.L. (2011). Polymerization of ɛ-caprolactone using ruthenium(II) mixed metallocene catalysts and isopropyl alcohol: living character and mechanistic study. J. Mol. Catal. A: Chem..

[11] Jitonnom J., Molloy R., Punyodom W., Meelua W. (2016). Theoretical studies on aluminum trialkoxide-initiated lactone ring-opening polymerizations: roles of alkoxide substituent and monomer ring structure. Comp. Theor. Chem..

[12] Karttunen V.A., Linnolahti M., Pakkanen T.A., Severn J.R., Kokko E., Maaranen J. (2008). Influence of the ligand structure of hafnocene polymerization catalysts: a theoretical study on ethene insertion and chain propagation. Organometallics.

[13] Karttunen V.A., Linnolahti M., Turunen A., Pakkanen T.A., Severn J.R., Maaranen J. (2008). The influence of the ligand structure on activation of hafnocene polymerization catalysts: a theoretical study. J. Organomet. Chem..

[14] Sattayanon C., Sontising W., Jitonnom J., Meepowpan P., Punyodom W., Kungwan N. (2014). Theoretical study on the mechanism and kinetics of ring-opening polymerization of cyclic esters initiated by tin(II) n-butoxide. Comp. Theor. Chem..

[15] Tanaka R., Nakayama Y., Shiono T. (2016). Theoretical investigation of the mechanism of syndiospecific propylene polymerization using ansa-dimethylsilylene(fluorenyl)(amido)titanium complexes. J. Organomet. Chem..

[16] Jitonnom J., Meelua W. (2017). Cationic ring-opening polymerization of cyclic carbonates and lactones by group 4 metallocenes: a theoretical study on mechanism and ring-strain effects. J. Theor. Comput. Chem..

